# Carbonized Leather Waste with Deposited Polypyrrole Nanotubes: Conductivity and Dye Adsorption

**DOI:** 10.3390/nano13202794

**Published:** 2023-10-19

**Authors:** Jaroslav Stejskal, Fahanwi Asabuwa Ngwabebhoh, Miroslava Trchová, Jan Prokeš

**Affiliations:** 1 University Institute, Tomas Bata University in Zlin, 760 01 Zlin, Czech Republic; asabuwa@utb.cz; 2 Central Laboratories, University of Chemistry and Technology, Prague, 166 28 Prague 6, Czech Republic; miroslava.trchova@vscht.cz; 3 Faculty of Mathematics and Physics, Charles University, 180 00 Prague 8, Czech Republic; jprokes@semi.mff.cuni.cz

**Keywords:** bicontinuous structure, carbonized leather, conducting polymer, globular polypyrrole, polypyrrole nanotubes, conductivity, resistivity, dye adsorption, Raman spectra

## Abstract

This paper reports the conversion of a waste to a conducting material, exploiting the ability to adsorb pollutant organic dyes. Leather waste was carbonized at 800 °C in an inert nitrogen atmosphere. The resulting biochar was used for in-situ deposition of polypyrrole nanotubes produced by the oxidative polymerization of pyrrole in the presence of methyl orange. The composites of carbonized leather with deposited polypyrrole nanotubes of various composition were compared with similar composites based on globular polypyrrole. Their molecular structure was characterized by infrared and Raman spectra. Both conducting components formed a bicontinuous structure. The resistivity was newly determined by a four-point van der Pauw method and monitored as a function of pressure applied up to 10 MPa. The typical conductivity of composites was of the order of 0.1 to 1 S cm^−1^ and it was always higher for polypyrrole nanotubes than for globular polypyrrole. The method also allows for the assessment of mechanical features, such as powder fluffiness. The conductivity decreased by 1–2 orders of magnitude after treatment with ammonia but still maintained a level acceptable for applications operating under non-acidic conditions. The composites were tested for dye adsorption, specifically cationic methylene blue and anionic methyl orange, using UV-vis spectroscopy. The composites were designed for future use as functional adsorbents controlled by the electrical potential or organic electrode materials.

## 1. Introduction

Novel functional materials are often based on the design of nanostructured composites, which display attractive properties and can be applied in new directions. The present study is oriented at conducting materials comprising organic nitrogen-containing carbon obtained by the carbonization of leather waste and conducting polymer, polypyrrole, with special attention paid to its nanotubular form. Both components conduct at semiconductor level, and while the former component is a product of an environmentally friendly process, the latter is a representative of responsive redox-active polymers. The fibrous biochar obtained from leather with conducting polymer deposited at its surface results in a bicontinuous structure that is favourable for conduction [1].

Bicontinuous composites composed of two interpenetrating phases have often been reported in the literature and concern typically inorganic systems. They display improved mechanical and electrical properties compared with simple mixtures of components. The term of three-dimensional interpenetrating matrices of two phases [2] has been used specially for inorganic combinations of ceramics, e.g., alumina/titanium aluminide [3], or ceramics combined with metals, e.g., silicon carbide/aluminium [4] or silica/aluminium [5]. Inorganic and organic components have also been combined and are represented by either fibrous inorganic component dispersed in an organic polymer matrix, e.g., silver nanowire network in polyurethane matrix [6], or, vice versa, by porous inorganics filled with organic polymers, e.g., alumina penetrated with polycarbonate [7].

Among the organic materials, bicontinuous composites should be distinguished from interpenetrating polymer networks where the three-dimensional structure is based on individual polymer chains and not by the phases composed of multiple polymer threads in the composites. From organic composites, melamine sponge coated with conducting polymers [8] or the fibrous collagen structure of leather modified in a similar manner may serve as examples [9].

Carbonization of leather waste to a fibrous conducting nitrogen-containing biochar, i.e., a waste to useful material, has been reported [10,11,12]. Applications involving this type of biochar concern dye adsorbents [13,14], removal of harmful chromium(VI) from wastewater [15,16], electromagnetic radiation shielding [11], supercapacitor electrodes [17,18], batteries [19,20] or fuels [21,22]. In some of them, electrical conduction is required; in others, it is of potential benefit.

The carbons derived from leather waste are produced as macroporous sponges with poor mechanical properties and as powder difficult to process after they are pulverized. They can be directly used as fillers in construction materials [23,24]. On the other hand, they can be converted to functional nanomaterials by coating with conducting polymers, such as polyaniline [1] or polypyrrole, as demonstrated in the present study. Such composites could be compressed to solid materials with applicable mechanical integrity and may benefit from the similar or complementary properties of both components (Table 1).

The present manuscript reports mainly electrical properties of polypyrrole composites with carbonized leather and illustrates their application for dye removal in water-pollution treatment. Although such use is not associated directly with conductivity, the adsorption control by the applied electrical potential is a route to the functional adsorbents.

## 2. Experimental

### 2.1. Preparation

Chrome-plated pigskin leather with 0.8 mm thickness was provided by the Footwear Research Centre, Tomas Bata University in Zlin, Czech Republic, and used for the simulation of the waste. Pyrrole, iron(III) chloride hexahydrate, methylene blue, methyl orange dyes and auxiliary chemicals were supplied by Sigma Aldrich (Prague, Czech Republic) and used as delivered.

Leather was shredded into fibres and carbonized in a horizontal tubular vacuum furnace GSL-1600X (Carbolite Gero, Neuhausen, Germany). During carbonization, the temperature increased at a rate of 5 °C min^−1^ to 800 °C under 50 mL min^−1^ argon flow. After 1 h, the power was switched off. The next day, the product was homogenized in a ball mill. It contained 70.7 wt% carbon, 7.6 wt% nitrogen and 12.6 wt% chromium [25].

**Figure 1:**
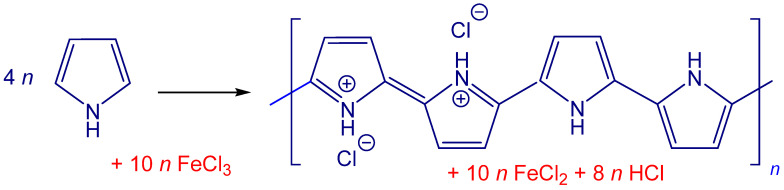
The oxidation of pyrrole with iron(III) chloride yields polypyrrole hydrochloride.

Polypyrrole coating was deposited in situ during the polymerization of pyrrole (Figure 1). Parts of the carbonized leather were dispersed in 0.2 M aqueous pyrrole solution under stirring at room temperature. The same volume of 0.5 M iron(III) chloride solution was added. The reaction mixture thus contained 0.1 M pyrrole and 0.25 M iron(III) chloride. Based on stoichiometry (Figure 1), 100 mL of mixture is expected to produce about 1 g of globular polypyrrole, but the true yield may be somewhat affected by the type and number of counter-ions. The volumes of reactant solutions were varied to obtain approximately 1 g of composites (Table 2). Polypyrrole nanotubes were deposited in a similar manner, although the reaction mixture additionally contained 0.005 M methyl orange. The dye was dissolved along with the pyrrole. After 1 h afforded for the pyrrole polymerization, dark precipitates were collected on a filter, well rinsed with 0.2 M hydrochloric acid, then with ethanol, and left to dry in air. A part of samples was deprotonated by overnight suspension in 1 M ammonium hydroxide.

### 2.2. Morphology

Scanning electron microscopy was performed using a Nova NanoSEM electron microscope (FEI, Brno, Czech Republic) to reveal the morphology of polypyrrole and its composite with carbonized leather. Prior to analysis, the materials were gold sputter-coated using a JEOL JFC 1300 Auto Fine coater (JEOL, Tokyo, Japan).

### 2.3. Spectroscopy

FTIR spectra were collected with a Nicolet 6700 spectrometer (Thermo Fisher Scientific, Waltham, MA, USA) using a reflective ATR extension GladiATR (PIKE Technologies, Fitchburg, WI, USA). Spectra were recorded in the 4000–400 cm^−1^ range at 4 cm^−1^ resolution; 64 scans and Happ–Genzel apodization were conducted. Raman spectra were recorded with a Thermo Scientific DXR Raman microscope (Thermo Fisher Scientific, Waltham, MA, USA) operating with a 780 nm laser line. The laser beam was focused at the 50× objective. The scattered light was analysed by a spectrograph with holographic gratings of 900 lines mm^−1^ and a 50 μm pinhole width. The acquisition time was 10 s with 10 repetitions. Care was taken to avoid the deprotonation of polypyrrole with the laser beam.

### 2.4. Electrical Properties

The resistivity was recorded by a four-point van der Pauw method with a lab-made press including a cylindrical glass cell with an inner diameter of 10 mm [8]. The powders were placed between a glass support and a glass piston carrying four wire electrodes at its perimeter. The set-up included a Keithley 220 current source, a Keithley 2010 multimeter and a Keithley 705 scanner with a Keithley 7052 matrix card (Keithley Instruments Inc., Cleveland, OH, USA). The pressure was recorded up to 10 MPa (=102 kg cm^−2^) with a L6E3 strain gauge cell (Zemic Europe BV, Etten-Leur, The Netherlands). The force was applied with an E87H4-B05 stepper motor (Haydon Switch & Instrument Inc., Waterbury, CT, USA). The sample thickness was monitored during the compression by a Mitutoyo ID-S112X dial indicator (Mitutoyo Corp., Sakado, Japan). The resistivity was separately determined on composite pellets prepared by compression at 527 MPa with a manual hydraulic press (Specac Ltd., Orpington, UK).

### 2.5. Dye Adsorption

The performance of composites in the removal of organic dyes from aqueous solutions was studied with UV-vis spectroscopy. The dye adsorption was followed in a batch reactor for 24 h at 25 °C, pH 5.5, initial dye concentration 100 mg L^−1^ and sample dosage 500 mg L^−1^. The adsorption was monitored by recording optical absorption with a Varian Cary 100 UV-vis spectrophotometer (Varian Inc., Palo Alto, CA, USA) in a 0.2 cm cell at absorption maxima 466 and 594 nm for methyl orange and methylene blue, respectively. The efficiency of dye removal was then evaluated as *R*(%) = (*A*_0_ − *A*_e_)/*A*_0_ × 100 [26], where *A*_0_ and *A*_e_ are the optical absorption at start and in equilibrium, respectively.

## 3. Results and Discussion

### 3.1. Preparation

**Figure 2:**
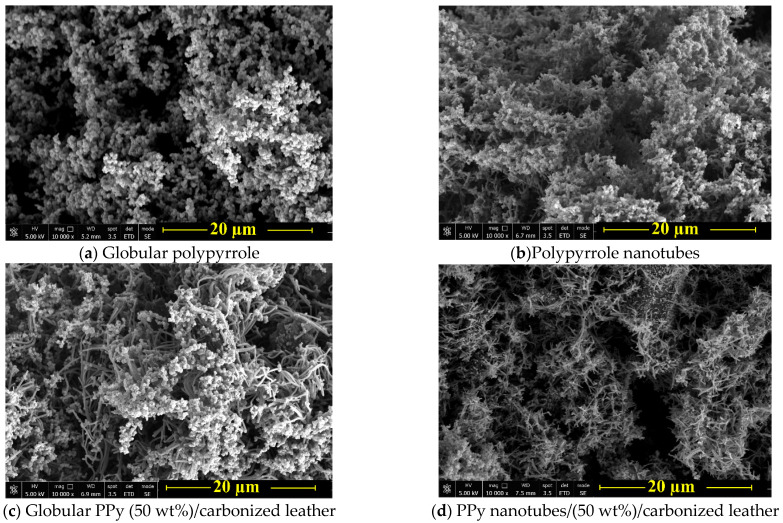
SEM micrographs of (**a**) globular polypyrrole, (**b**) polypyrrole nanotubes and (**c**,**d**) their composites after the deposition of polypyrrole on carbonized leather. For neat carbonized leather, see Ref. [12].

Polypyrrole is typically prepared by the oxidation of pyrrole in an aqueous medium, iron(III) chloride being the most popular oxidant [27] (Figure 1). The product has a characteristic globular morphology (Figure 2). It has been observed by transmission electron microscopy that polypyrrole nanotubes are produced instead when the polymerization is carried out in the presence of methyl orange [28,29]. The change in morphology is associated with the increase in conductivity by about one order of magnitude. Other organic dyes are also able to affect the morphology and conductivity of polypyrrole [30], but methyl orange performs the best.

Any substrate immersed in the reaction medium used for the polymerization of pyrrole or aniline becomes coated with a submicrometre film of the polymer, and polymer nanoparticles adhere to its surface [8,9,31,32,33]. When it comes to the resulting composites, the composite morphology becomes more complex (Figure 2). In the absence of methyl orange, polypyrrole globules adhere on the polypyrrole-coated carbonized collagenous fibres constituting the original leather. The morphology becomes more uniform when in the presence of methyl orange, as the well-developed polypyrrole nanotubes completely coat the carbonized leather fibres.

### 3.2. Spectroscopy

**Figure 3:**
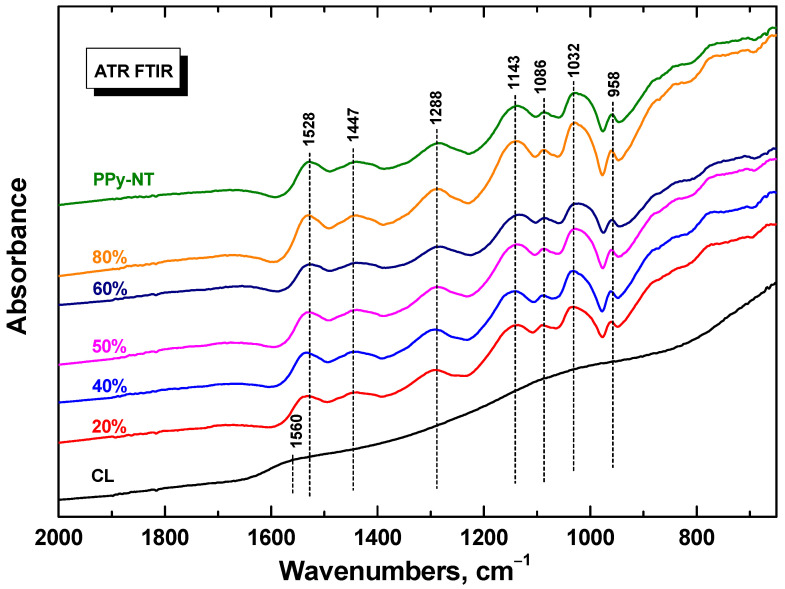
ATR FTIR spectra of carbonized leather (CL) coated with various amount of polypyrrole nanotubes (PPy-NT).

FTIR spectra of polypyrrole do not depend on the polypyrrole morphology and only the spectra of composites with polypyrrole nanotubes are demonstrated (Figure 3). The infrared spectrum of carbonized leather is practically featureless. After coating with polypyrrole nanotubes we observe the main bands of polypyrrole due to the surface sensitive nature of the ATR spectroscopic technique with a penetration depth of a few micrometres of infrared radiation. They are situated at 1528 cm^−1^ (C–C stretching vibrations in the pyrrole ring), 1447 cm^−1^ (C–N stretching vibrations in the ring), 1288 cm^−1^ (C–H or C–N in-plane deformation modes), 1143 cm^−1^ (breathing vibrations of the pyrrole rings), 1032 cm^−1^ (C–H and N–H in-plane deformation vibrations) and 958 cm^−1^ (C–H out-of-plane deformation vibrations of the ring) [29,34].

**Figure 4:**
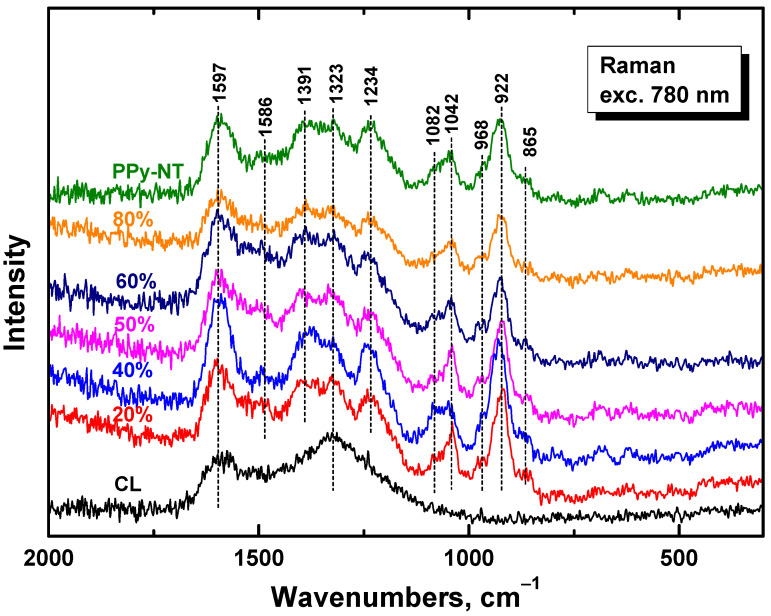
Raman spectra of carbonized leather (CL) coated with various amounts of polypyrrole nanotubes (PPy-NT).

In the Raman spectrum of carbonized leather, we detect the two main bands with local maxima at 1597 and 1323 cm^−1^ characteristic of carbon-like material [35] (Figure 4). After coating with polypyrrole nanotubes we mainly observe the bands of polypyrrole, due to the resonance of the energy of the laser excitation wavelength 780 nm with the energy of polarons in the polypyrrole salt. This also confirms the complete coating of biochar with polypyrrole. The bands are situated at 1597 cm^−1^ and 1586 cm^−1^ (C=C stretching vibrations of polypyrrole backbone), 1391 and 1323 cm^−1^ (two bands of ring-stretching vibrations, the intensity of the latter increasing after deprotonation) and 1234 cm^−1^ (antisymmetric C–H deformation vibrations). The double peak with local maxima at 1082 and 1042 cm^−1^ corresponds to C−H out-of-plane deformation vibrations (the second becomes sharper after deprotonation). The band at 968 cm^−1^ of the ring-deformation vibrations in neutral polypyrrole units and the sharp peak at 922 cm^−1^ of the ring-deformation vibrations in dication (bipolaron) units are also detected in the spectra [29].

### 3.3. Electrical Properties

Both composite components are conducting at semiconductor level (Table 1). Some materials are difficult to compress to self-standing pellets used for the routine determination of resistivity. For that reason, the resistivity has to be determined on powder compressed under defined pressure. This quantity becomes pressure-dependent and the resistivity decreases during compression [36,37]. The present study thus uses a four-point method [8], which is preferred to the two-point procedure because of the relatively high conductivity of samples.

**Figure 5:**
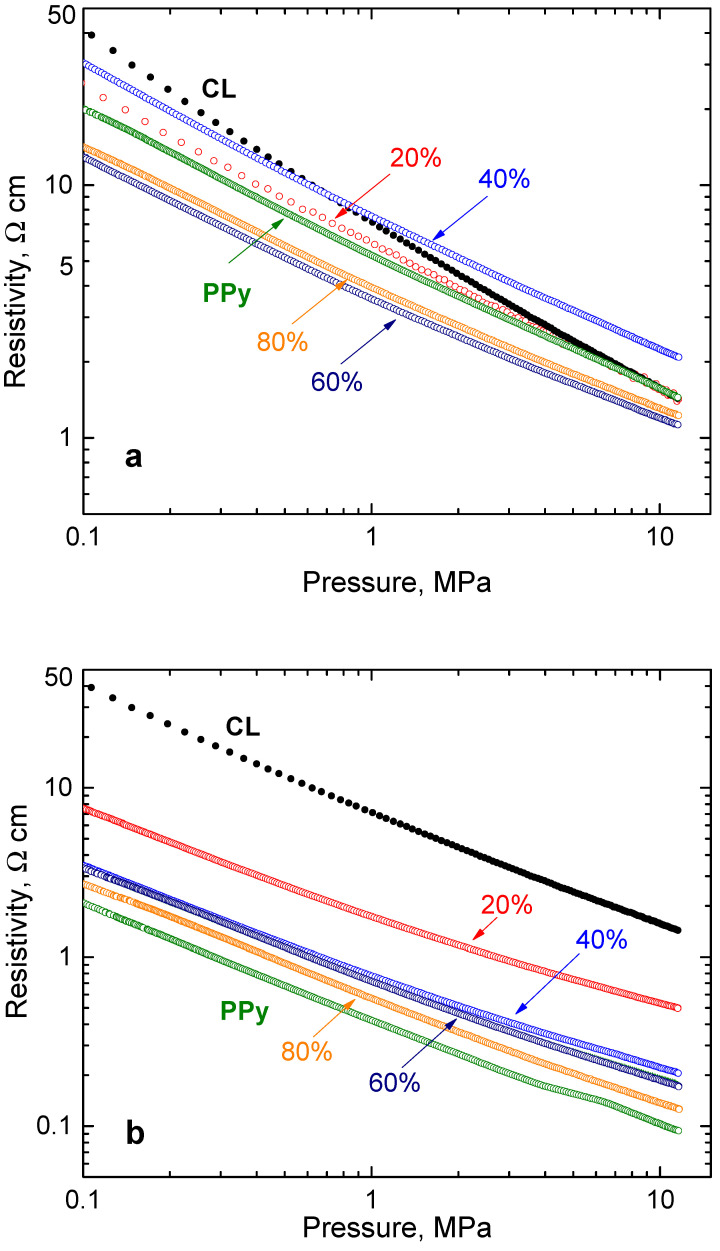
The pressure dependence of the resistivity of leather carbonized at 800 °C (CL) coated with various amounts of (**a**) globular polypyrrole (PPy-G) and (**b**) polypyrrole nanotubes (PPy-NT).

Carbonized leather has a resistivity of the same order of magnitude as globular polypyrrole [12], and the same applies for their composites (Figure 5a). The analogous samples with polypyrrole nanotubes are more conducting, their resistivity being lower by approximately one order of magnitude (Figure 5b). The pressure dependences of resistivity are linear in double-logarithmic presentation in both cases, and for all composites they have a similar slope. The dependence for neat carbonized leather is steeper in the accordance with the different mechanical properties (cf. below).

**Figure 6:**
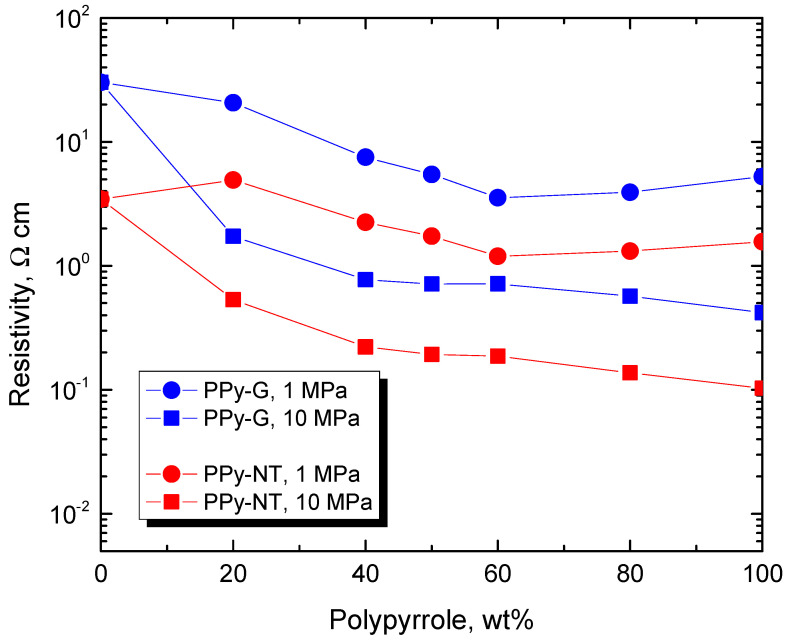
Resistivity of composites of globular polypyrrole or polypyrrole nanotubes and carbonized leather compressed at 1 MPa and 10 MPa.

The resistivity of the individual samples at 1 and 10 MPa pressures are available in Figure 6, and they provide the rather trivial information that (1) the resistivity varies little with the composition, (2) it becomes lower at higher pressure and (3) composites with polypyrrole nanotubes have significantly lower resistivity compared to those with globular polypyrrole. For readers preferring the presentation in terms of conductivity rather than reciprocal resistivity, some key values are provided in Table 3.

### 3.4. Mechanical Properties

**Figure 7:**
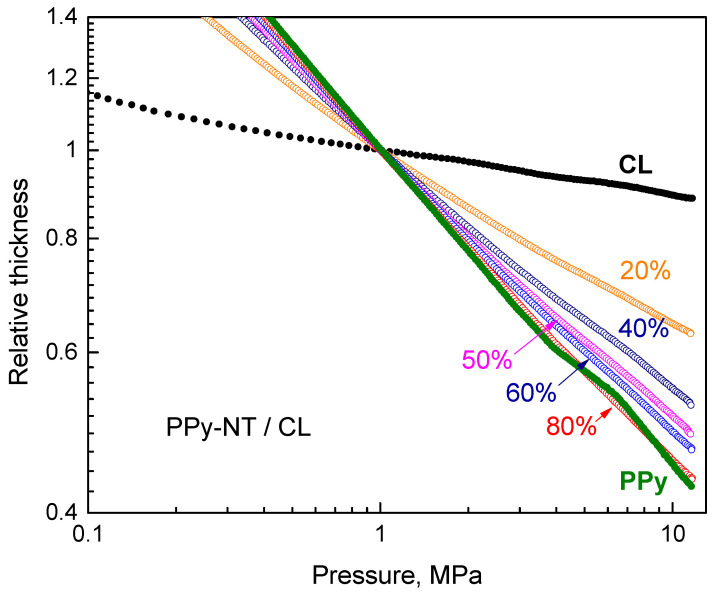
The change of sample thickness during the compression normalized to the thickness at 1 MPa.

The experimental setup used for the determination of resistivity also allows to follow the sample thickness during the compression [8], i.e., to also assess some features of mechanical behaviour. They were similar for both types of polypyrrole and only the results concerning the deposition of polypyrrole nanotubes are reported (Figure 7). The double-logarithmic dependences of sample thickness on pressure are close to linear for all composites. The steeper the dependences are, the fluffier the composites are and the easier they are compressed. The absolute values of slopes decrease slowly as the content of polypyrrole increases. The carbonized leather alone is the most difficult to compress and resembles inorganic rather than organic materials.

### 3.5. Deprotonation

**Figure 8:**
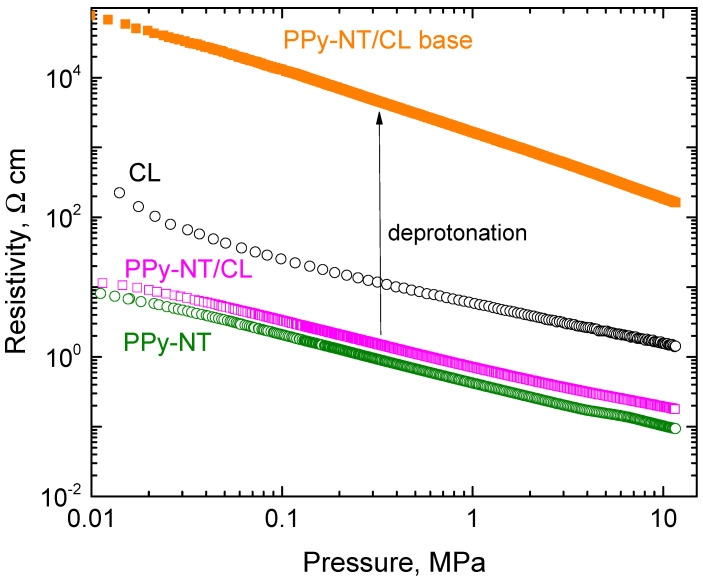
The pressure dependences of resistivity of polypyrrole nanotubes (PPy-NT) and their composites (50 wt% PPy) with carbonized leather and the effect of composite deprotonation.

For some applications, e.g., in biomedicine, the effect of pH may become of importance. The conductivity of carbonized leather is not affected by pH [1]. The conductivity of polypyrrole, however, is reduced in alkaline ammonia solution due to the deprotonation of polypyrrole salt (Figure 1) to a less conducting base [38], but the polymer still retains most of its conductivity. Similar behaviour applies to the composite with carbonized leather. For example, the conductivity of polypyrrole nanotubes (50 wt%) with carbonized leather was reduced after treatment with ammonia solution by about two orders of magnitude (Figure 8), from 16.1 S cm^−1^ to 0.0195 S cm^−1^ when measured on a compressed pellet; i.e., a good level of conductivity was maintained regardless of non-acidic pH treatment.

### 3.6. Dye Adsorption

One of the application areas shared by microstructured carbons [39,40,41] and conducting polymers [42,43,44] is the adsorption of water pollutants. The removal of organic dyes from wastewater is based on the adsorption and/or the photocatalytic decomposition or by biodegradation [45,46]. Electrocatalytic dye destruction employing conducting polymers is also possible [47,48]. There are two reasons for introducing polypyrrole to hybrid composites used as adsorbents: (1) Conducting polymers and dyes share features of molecular structure, i.e., the presence of nitrogen atoms and aromatic rings. In addition to dispersion forces, nitrogen atoms participate in hydrogen bonding and the benzene rings allow for the attractive π–π interactions supporting the adsorption. Electrostatic interactions between polypyrrole polycation (Figure 1) and anionic dyes may also be operational. (2) In the contrast to carbonaceous adsorbents, conducting polymers are electroactive and responsive, i.e., they respond to external stimuli by a change in electrical properties. The molecular structure of conducting polymers can be converted between protonated salt and neutral base, as well as between oxidized and reduced structures, in response to pH change or electrical potential, respectively [49,50].

**Figure 9:**
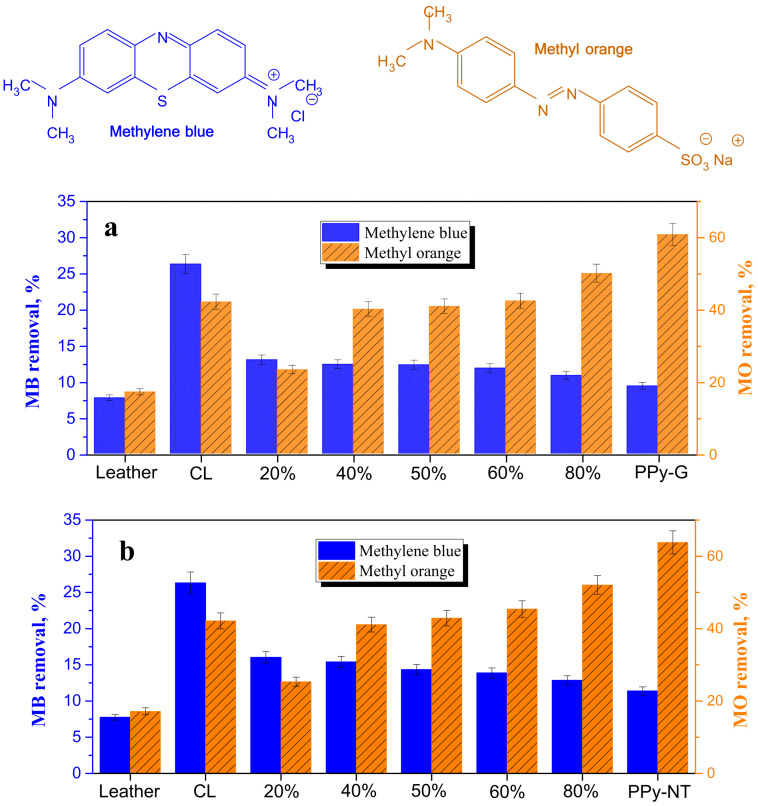
Removal of dyes, methylene blue and methyl orange, with leather, carbonized leather and composites of carbonized leather with different percentages of polypyrrole (**a**) globules and (**b**) nanotubes. N.B.: The scales for both dyes are different.

The dye removal by the composites was tested in the present study with cationic methylene blue and anionic methyl orange dyes (Figure 9). The adsorption performance of methylene blue and methyl orange on the individual composite components has been reported in the literature. The carbonized leather wastes have been applied mainly for the adsorption of a cationic dye, methylene blue (Basic Blue 9) [10,51,52,53,54,55,56]. The adsorption of methyl orange (Acid Orange 52) has only been reported seldom [1,12] and was found to be better compared with methylene blue. All papers report the dye adsorption but quantitative comparison is not possible due to the variations in concentrations of dyes and adsorbents and the general experiment design. The reports also concern leathers of various origin and different ways used for carbonization and activation.

The adsorption of methylene blue on polypyrrole has also been reported [57,58,59,60,61,62] but the comparison of results is again difficult, due also to variable polymer morphology depending on the polymerization conditions. Polypyrrole was not investigated alone but only in composites with inorganic species. The removal of methyl orange was reported only exceptionally [63].

The present results (Figure 9) are only an illustration of adsorption feasibility and experiments have not been optimized with respect to dye and composite concentrations or other parameters. While the dye adsorption of the original leather was marginal, it improved for both dyes after carbonization. The adsorption of methylene blue was lower compared with methyl orange and it slowly decreased with increasing content of polypyrrole in the composites. On the other hand, the adsorption of methyl orange had an increasing trend, as may be expected for an anionic dye at polypyrrole polycation. Similar adsorption performance has been found regardless of polypyrrole morphology (Figure 9).

## 4. Conclusions

The carbonized leather was coated with polypyrrole during the polymerization of pyrrole. The resistivity of the resulting composite powders decreased during compression up to 10 MPa. The conductivity of the composites was little dependent on the polypyrrole content in the composites and was of the order of 0.1 S cm^−1^ for globular polypyrrole and 1 S cm^−1^ for polypyrrole nanotubes at 10 MPa. The conductivity of the composites compressed to pellets was still about one order of magnitude higher. The conductivity decreased by approximately two orders of magnitude after deprotonation of polypyrrole with ammonia but maintained a level acceptable for applications operating under alkaline or close to neutral physiological conditions. When tested for dye adsorption, the composites rich in carbonized leather better adsorbed a cationic dye, methylene blue, while those containing more polypyrrole preferentially removed an anionic dye, methyl orange, from aqueous media regardless of polypyrrole morphology. While most of the properties of the composites based on globular and nanotubular polypyrrole are comparable, those with polypyrrole nanotubes are the most prospective due to their higher conductivity. The composites might be suitable for further studies of dye adsorption influenced or controlled by applied electrical potential.

## Figures and Tables

**Table 1:** Properties of the composite components, carbonized leather and polypyrrole [8,10,11,12,13,14].

Property	Carbonized Leather	Polypyrrole
Conductivity	10^−8^–10^0^ S cm^−1^	10^−1^–10^1^ S cm^−1^
pH Sensitivity of conductivity	none	reduced above pH 4–6
Thermal stability	stable	converts to carbon
Molecular structure	carbonaceous, graphitic	conjugated polymer chains
Supramolecular structure	fibrous	globules or nanotubes
Specific surface area	activation dependent	low
Redox activity	none	yes
Dye adsorption	yes	yes

**Table 2:** The protocol for the preparation of approximately 1 g of composites composed of polypyrrole (PPy) and carbonized leather with gradually increasing polypyrrole content, and the experimental yield.

Composition, wt% PPy	Carbonized Leather, g	+0.2 M Pyrrole, mL	+0.25 M FeCl_3_, mL	PPy, g	Yield, g
20	0.8	10	10	0.2	1.041
40	0.6	20	20	0.4	1.102
50	0.5	25	25	0.5	1.181
60	0.4	30	30	0.6	1.266
80	0.2	40	40	0.8	1.351
100	0	50	50	1	1.543

**Table 3:** Conductivity (S cm^−1^) of leather carbonised at 800 °C (CL), globular polypyrrole (PPy-G) or polypyrrole nanotubes (PPy-NT) and their composites (50 wt%) determined at 1 or 10 MPa pressure or with free-standing compressed pellet (527 MPa).

Sample	1 MPa	10 MPa	Pellet
CL	0.269	3.71	(a)
PPy-G	0.190	0.637	2.55
PPy-NT	2.38	9.71	38.6
PPy-G/CL	0.183	0.575	1.96
PPy-NT/CL	1.40	5.18	16.1

(a) A pellet could not be prepared.
